# An Investigation into the Application of Acceleration Responses’ Trendline for Bridge Damage Detection Using Quadratic Regression

**DOI:** 10.3390/s24020410

**Published:** 2024-01-09

**Authors:** Hadi Kordestani, Chunwei Zhang, Ali Arab

**Affiliations:** 1 School of Civil Engineering, Shandong Jianzhu University, Jinan 250101, China; hadikordestani@zju.edu.cn; 2 Multidisciplinary Center for Infrastructure Engineering, Shenyang University of Technology, Shenyang 110870, China

**Keywords:** quadratic regression, trendline, damage detection, bridge, acceleration response, truckload

## Abstract

It has been proven that structural damage can be successfully identified using trendlines of structural acceleration responses. In previous numerical and experimental studies, the Savitzky–Golay filter and moving average filter were adjusted to determine suitable trendlines and locate structural damage in a simply supported bridge. In this study, the quadratic regression technique was studied and employed to calculate the trendlines of the bridge acceleration responses. The normalized energies of the resulting trendlines were then used as a damage index to identify the location and severity of the structural bridge damage. An ABAQUS model of a 25 m simply supported bridge under a truckload with different velocities was used to verify the accuracy of the proposed method. The structural damage was numerically modeled as cracks at the bottom of the bridge, so the stiffness at the damage positions was decreased accordingly. Four different velocities from 1 m/s to 8 m/s were used. The proposed method can identify structural damage in noisy environments without monitoring the dynamic modal parameters. Moreover, the accuracy of the newly proposed trendline-based method was increased compared to the previous method. For velocities up to 4 m/s, the damage in all single- and multiple-damage scenarios was successfully identified. For the velocity of 8 m/s, the damage in some scenarios was not located accurately. Additionally, it should be noted that the proposed method can be categorized as an online, quick, and baseline-free structural damage-detection method.

## 1. Introduction

A bridge is an infrastructure usually designed to have a long service life (more than normal buildings). Compared with normal buildings that face almost no changes in their service load, bridges gradually experience more loads due to the increased population and number of cars in society. Therefore, it is important to have a periodic assessment program to ensure bridge safety. A periodic bridge assessment program can identify possible structural damage in its early stages. In between, vibration-based bridge health monitoring has received extensive attention for decades.

Recording the acceleration response of a structure is an easy task. Accelerometers are relatively cheap and easy to install and use. Compared with displacement meters, they receive fewer effects from pseudo-static responses and better show the dynamic modal parameter, especially in higher dynamic modes. This study uses bridge acceleration responses and mostly addresses the methods that only use acceleration responses as input for their damage-detection method.

Various techniques (methods) have been proposed to process structural vibration responses recorded along the bridge under truckloads to locate structural damage. Some of the most important ones that could work with acceleration responses are transformer-based methods (such as the Wavelet transformer or Hilbert–Huang transformer) [1,2,3,4,5,6,7,8], blind source separation-based methods [9,10,11,12,13,14], and special averaging-based methods (e.g., random decrement technique) [15,16,17,18,19,20]. Although these techniques (methods) are very popular, they have difficult-to-understand mathematical backgrounds. Hence, the resulting damage-detection methods are also considered time-consuming and expensive. Based on the methods mentioned above (and the other damage-detection methods not addressed in this paper), several review papers discuss the benefits, challenges, and limitations of the vibration-based damage-detection method, such as [21,22,23,24,25,26,27,28,29].

A trendline of a signal is easy to understand and calculate. An easy way to determine a trendline for a signal is to use a simple moving average filter (sliding average). It has been proven that special trendlines calculated from acceleration responses can be used as inputs for damage-detection methods. Two moving average-based bridge damage-detection methods were proposed to utilize trendlines of acceleration responses and locate bridge damage [30,31]. The proposed methods were supported by numerical and experimental models of simply supported bridges under truckloads with different velocities. Later, Kordestani et al. [32,33] employed the Savitzky–Golay filter, determined more advanced trendlines for bridge acceleration responses, and successfully identified the location and severity of bridge damage. A complete experimental algorithm for signal decomposition using trendlines calculated by the Savitzky–Golay filter was addressed in [34]. This algorithm was then utilized to make a damage-detection method in [35,36]. Various experimental and numerical examples of bridges and buildings under different excitation loads were addressed in the above literature.

This paper aims to study the use of the quadratic regression technique (QRT) and increase the accuracy of the trendline-based bridge damage-detection method. Therefore, employing QRT, an output-only trendline-based damage-detection method is proposed to identify bridge structural damage under truckloads with different velocities. A numerical model of a simply supported bridge under a moving sprung mass is then used to verify the proposed method. It is proven that the proposed method can identify the structural damage of the bridge without the need to monitor the dynamic modal parameters. The proposed method is fast, accurate, and can be categorized as a quick, online, and baseline-free bridge damage-detection method.

## 2. Research Significance

The idea of using the acceleration’s trendline to fully decompose an acceleration signal was initially proposed in 2020 by [32,33,34]. The authors numerically and experimentally proved that the trendline of a structural acceleration response has signatures of structural dynamic behavior and damage. Later, further research on this idea was conducted to improve the accuracy of the proposed idea and provide more numerical and experimental evidence for it. Since, in this idea, it is essential to study the methods that calculate and determine trendlines for an acceleration response, the current work explores the use of quadratic regression as a powerful method to calculate more suitable trendlines for acceleration responses.

## 3. Quadratic Regression Technique

**Figure 1:**
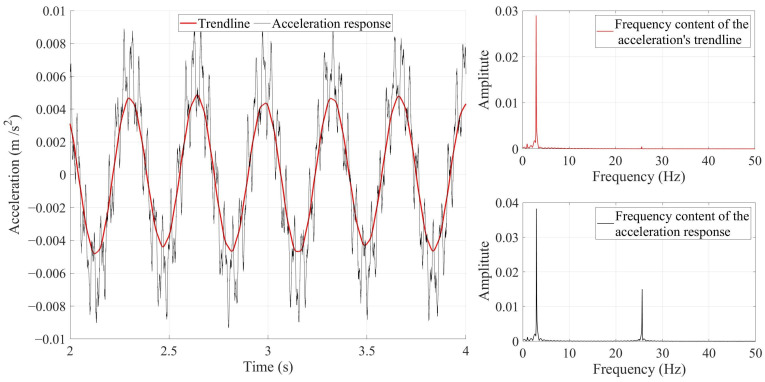
The effect of applying a well-adjusted Savitzky–Golay filter on a signal. The black color refers to the original signal. The red color refers to the trendline (filtered signal). The original signal and its trendline are plotted on the left side of the figure, and the fast Fourier transform of the original signal and its trendline are placed on the right side of the figure.

Smoothing techniques such as the Savitzky–Golay filter, Gaussian filter, or moving average filter are broadly used to attenuate noises in a signal. Kordestani et al. [33] proved that a well-adjusted Savitzky–Golay filter could determine a special trendline that mainly composes the first natural frequency of the bridge. They claimed that to have such a trendline, the span of the Savitzky–Golay filter must be set to (sampling frequency/first natural frequency). The bridge model used in this study has a first natural frequency equal to 2.93 Hz. The sampling frequency is 2000 data per second. Figure 1 shows the reason why the Savitzky–Golay filter acts like a suitable band-pass filter.

Figure 1, as an example, shows the acceleration signal recorded in the middle of the simply supported bridge under a moving sprung mass. The black color in Figure 1 refers to the acceleration response, and the red color refers to its trendline calculated using the Savitzky–Golay filter. Figure 1 shows the response’s fast Fourier transform (FFT) and trendline. As seen in Figure 1, the red FFT curve was weakened by the Savitzky–Golay filter, but they both have the same amplitude at 2.93 Hz (the third natural frequency vanishes from the curve). Even with the naked eye, it can be seen from Figure 1 that the trendline only consists of a unique natural frequency. Figure 1 proves that the well-adjusted Savitzky–Golay filter keeps the first natural frequency but attenuates the other natural frequencies and noises. The subsequent natural frequency in this figure is 25.5 Hz, which is the third bridge natural frequency. Since the second natural frequency of the bridge cannot be recorded in the middle of the bridge, it cannot be seen here.

Although the Savitzky–Golay filter works well, it is found that QRT can increase the accuracy of the determined trendlines. QRT considers the below formula to determine a fit for a data set.

(1) $$y=ax^{2}+bx+c,\;a\neq 0$$

In Equation (1), if parameter a=0, then it will be a simple regression technique. The least square method is usually employed to solve Equation (1). Therefore, considering each acceleration record as $\left(x_{i},y_{i}\right)$, for each $y_{i}$, there will be a $y=a{x_{i}}^{2}+bx_{i}+c$ as well. To find the best values for the parameters a, b, and c, the parameter R in the following equation should be close to the value of 1.

(2) $$R^{2}=1-\frac{\sum_{i=1}^{n}{\left(y_{i}-\left(a{x_{i}}^{2}+bx_{i}+c\right)\right)}^{2}}{\sum_{i=1}^{n}{\left(y_{i}-\bar{y}\right)}^{2}}$$

where $\bar{y}$ in Equation (2) is the mean of all $y_{i}$. $R^{2}$ is assumed to have a value between 0 and 1 in which the closer the value to 1, the better the curve fits. Hence, R→1, which means the calculated fit represents the data better. In Equation (2), the parameter n is the amount of data around record $x_{i}$ that needs to be considered for fit calculation. That means for each record, there is a need to calculate a separate fit. The final trendline will be $\left(x_{i},y\right)$. One way to solve Equation (2) is to consider the following steps.

(3) $$\bar{x}=\frac{1}{n}\sum_{i=1}^{n}x_{i},\;\bar{x^{2}}=\frac{1}{n}\sum_{i=1}^{n}{x_{i}}^{2},\;\bar{y}=\frac{1}{n}\sum_{i=1}^{n}y_{i}$$

(4) $$S_{xx}=\sum_{i=1}^{n}{\left(x_{i}-\bar{x}\right)}^{2},\;S_{xy}=\sum_{i=1}^{n}\left(x_{i}-\bar{x}\right)\left(y_{i}-\bar{y}\right)\;S_{xx^{2}}=\sum_{i=1}^{n}\left(x_{i}-\bar{x}\right)\left({x_{i}}^{2}-\bar{x^{2}}\right),\;S_{x^{2}x^{2}}=\sum_{i=1}^{n}{\left({x_{i}}^{2}-\bar{x^{2}}\right)}^{2}$$

$$S_{x^{2}y}=\sum_{i=1}^{n}\left({x_{i}}^{2}-\bar{x^{2}}\right)\left(y_{i}-\bar{y}\right)$$

Additionally, the best values for the parameters a, b, and c are:

(5) $$b=\frac{S_{xy}S_{x^{2}x^{2}}-S_{x^{2}y}S_{xx^{2}}}{S_{xx}S_{x^{2}x^{2}}-{S_{xx^{2}}}^{2}}\;a=\frac{S_{x^{2}y}S_{xx}-S_{xy}S_{xx^{2}}}{S_{xx}S_{x^{2}x^{2}}-{S_{xx^{2}}}^{2}}\;c=\bar{y}-b\bar{x}-a\bar{x^{2}}$$

**Figure 2:**
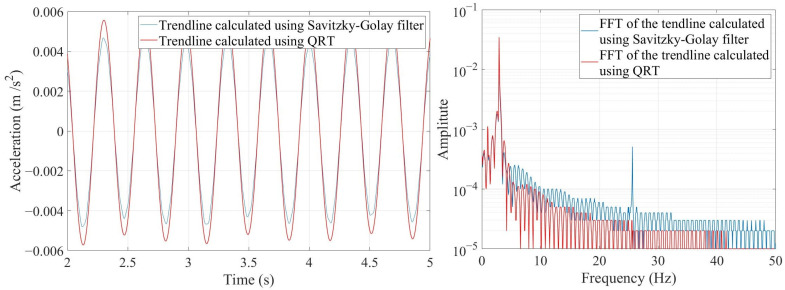
Comparison between the trendlines calculated using the Savitzky–Golay filter and QRT and their frequency contents.

Many software applications such as “Signal Analyzer Toolbox in MATLAB R2021b” are available to calculate the above formula and determine the acceleration’s trendline. Figure 2 compares the trendlines calculated using the Savitzky–Golay filter and QRT.

**Figure 3:**
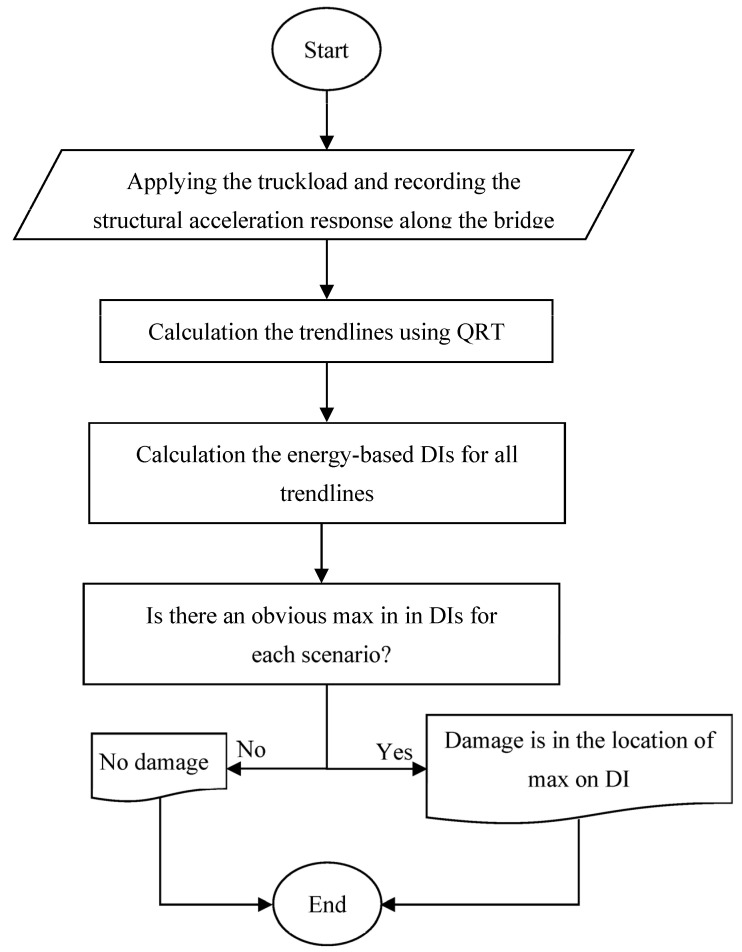
Flowchart of the proposed method.

As shown in Figure 2, the trendlines calculated using both methods are almost the same, but their frequency contents are a bit different. For example, the third natural frequency is completely vanished and, except for the first natural frequency, the amplitude of the rest of the frequencies is lower when QRT is utilized. Therefore, compared with the Savitzky–Golay filter, the QRT identifies a better trendline regarding frequency content for a signal. It should be noted that the resulting trendlines represent the dynamic behavior of a structure in a specific natural frequency. The flowchart of the proposed method is shown in Figure 3.

## 4. Numerical Simulation of a 25 m Simply Supported Bridge Subjected to a Truckload

**Figure 4:**
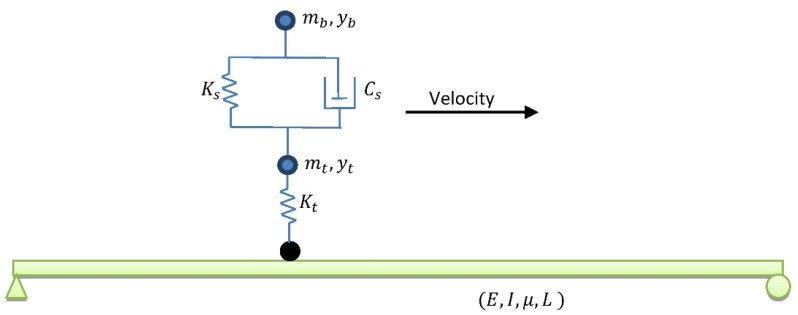
Schematic view of the simply supported bridge under a truckload.

A simply supported bridge under a truckload is usually used to verify the accuracy of most bridge damage-detection methods initially [33,36,37]. Although a truckload in such a simulation can be defined as 1—moving concentrated load, 2—moving mass, and 3—moving sprung mass, a theoretical study proved that the moving sprung mass could show a better simulation for a truckload [38]. Moreover, shifts in bridge natural frequencies due to the interaction between the bridge and vehicle can also be simulated when the moving sprung mass is utilized [39,40,41]. Therefore, a numerical model of a simply supported bridge with a length of 25 m under a moving sprung mass with different velocities is considered in this paper. A schematic view of this bridge is shown in Figure 4. The details of the bridge and truckload are listed in Table 1 and Table 2. It should be noted that the rotational degree of freedom of the moving sprung mass is restricted in this simulation.

**Figure 5:**
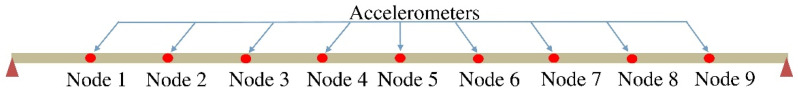
Schematic location of where the acceleration responses were obtained along the bridge.

ABAQUS software 6.14 was utilized to establish the FEM model of bridge–vehicle simulation. In the first step, gravity was applied to the FEM model. In the second step, the moving sprung mass moves along the simply supported bridge, and the bridge acceleration responses were recorded at fixed distance positions with a sampling frequency of 2000 data per second. The full Newton algorithm was utilized to perform the static and dynamic analysis in the FEM model. The moving sprung mass was modeled using three reference points and two springs, which can only vibrate vertically when they move along the bridge with constant velocity. The mass of the vehicle body and axle is assigned to the top two reference points. Frictionless hard contact was used as an interaction type between the bridge and the third reference point. The low-damped system has been reported for many simply supported bridges in the literature [30,42], and therefore, the bridge’s damping was also ignored in this simulation. However, the moving sprung mass has a damper as mentioned in Table 2. Figure 5 shows the uniformly distributed nodes along the bridge where the acceleration responses were obtained. Additionally, five damage scenarios were also introduced to the bridge, as listed in Table 3.

## 5. Results and Discussion

**Figure 6:**
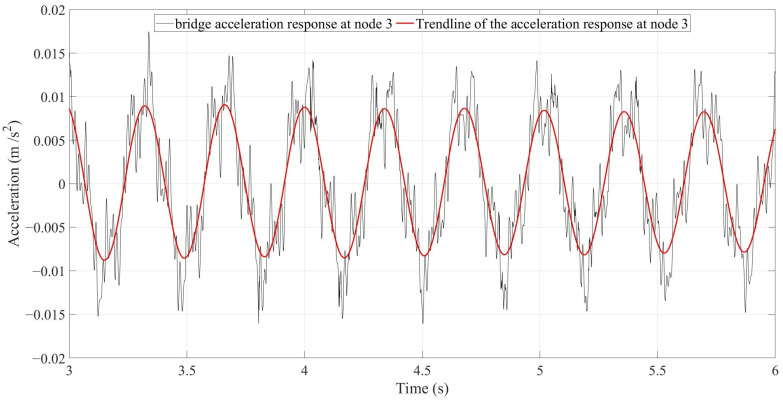
The black signal is the acceleration response of the bridge recorded at Node 3 under a truckload with a velocity of 2.5 m/s. The red signal is the trendline of the acceleration response calculated using QRT.

As mentioned above, there are four single-damage scenarios, one multi-damage scenario, and an undamaged scenario. As a result, there are a total number of six scenarios. Since four different velocities were defined for the vehicle in this study, 24 simulations were run accordingly. To explain the result, the bridge acceleration response of Node 3 in the undamaged scenario with its trendline calculated using QRT are shown in Figure 6.

There are 24 scenarios and 9 recorded acceleration data for each scenario. Hence, 216 trendlines were calculated for this study. A normalized energy-based damage index (NDI) should be defined and calculated for each trendline in the next step.

### 5.1. Normalized Energy-Based Damage Index

The energy of a trendline can be calculated using the formula below.

(6) $$E=\int {\left(Tr\right)}^{2}dt$$

where E is the energy and Tr stands for the trendline calculated using QRT. Obviously, any change in the scenarios or velocities ends with a change in the E. To normalize the parameter E, a normalization factor is defined below:

(7) $$\gamma_{i}=\frac{{E_{IN}}_{i}}{\bar{E_{IN}}}$$

In Equation (7), $\gamma_{i}$ is the normalization factor for node i. Index “IN” refers to the undamaged scenario (intact scenario) and therefore ${E_{IN}}_{i}$ is the energy of the trendline at node i for the undamaged scenario. The $\bar{E_{IN}}$ is the average of all ${E_{IN}}_{i}$. In the next step, the NDI is calculated using the following formula:

(8) $${NDI}_{i}=\left(\frac{E_{i}}{\bar{E}}\times \frac{100}{\gamma_{i}}\right)-100$$

In Equation (8), $E_{i}$ is the energy of the trendline of acceleration response at node i for the damaged scenario. $\bar{E}$ is the mean of $E_{i}$. NDI for an undamaged scenario causes all nodes in Figure 5 to show a normalized energy equal to zero. If a bridge experiences structural damage, the energy distribution along the bridge also changes. Using the NDI, it is supposed to have a peak in the vicinity of the structural damage.

### 5.2. Locating Bridge Structural Damage

**Figure 7:**
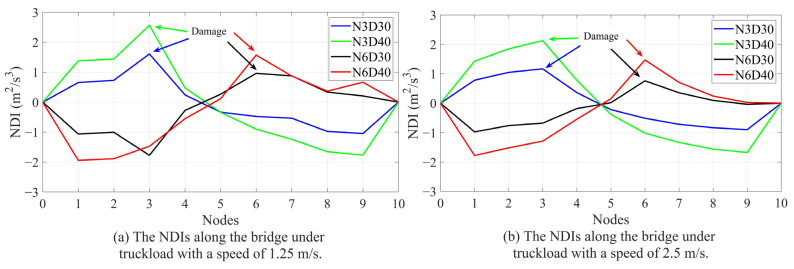

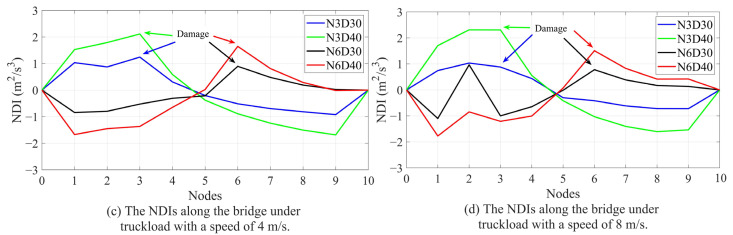
The NDIs along the bridge of different scenarios and different velocities: (**a**) velocity = 1.25 m/s, (**b**) velocity = 2.5 m/s, (**c**) velocity = 4 m/s, (**d**) velocity = 8 m/s.

**Figure 8:**
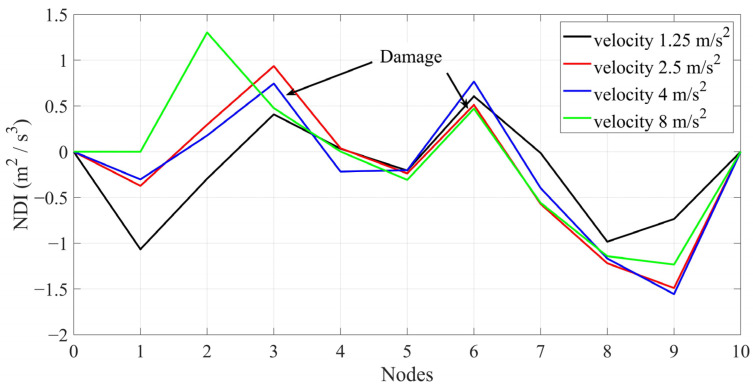
The NDIs along the bridge of multi-damage scenario and different velocities.

As mentioned above, there should be a maximum in the NDI in the vicinity of structural damage. Figure 7 shows the NDI for each scenario (single damage) and velocity. Figure 8 shows the multi-damage scenario and different velocities. Figure 7 and Figure 8 show that the maximum number of NDIs in each scenario can easily be detected except for a velocity of 8 m/s. Hence, the accuracy of the proposed method will decrease by increasing the vehicle’s velocity. The results shown in Figure 7 and Figure 8 are from noise-free environments.

### 5.3. Noise Consideration

**Figure 9:**
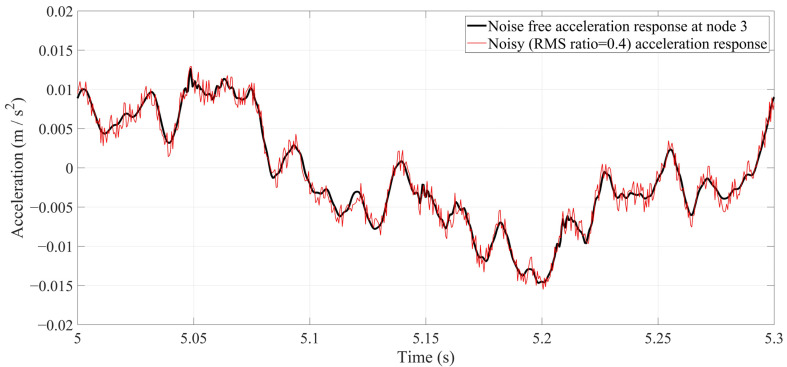
Acceleration response at Node 3 under a truckload with a speed of 2.5 m/s. The black color is a noise-free response, and red color is a noisy response (RMS ratio = 0.4).

**Figure 10:**
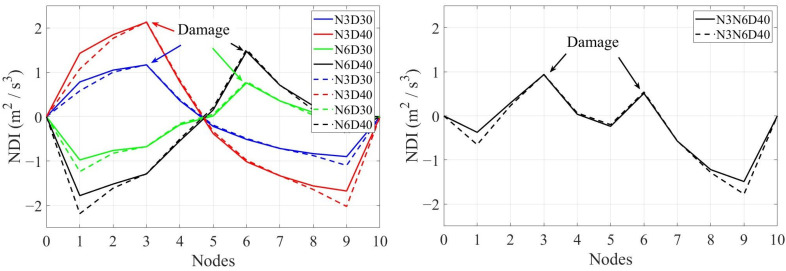
Acceleration response at Node 3 under a truckload with a speed of 2.5 m/s. Solid lines are noise-free responses, and dashed lines are noisy responses (RMS ratio = 0.4).

It is essential to bring a damage-detection method into real practice. However, for this study, there was no access to an experimental model or real bridge. When there is no access to an experimental model, different levels of white noise can be considered to simulate real-world conditions. To do this, three levels of white noise were generated and manually added to all acceleration responses. The root mean square (RMS) ratio was used to determine the noise level of the signal. Three levels of RMS ratio, namely 0.1, 0.25, and 0.4, were considered. Figure 9 shows the noise level of the RMS ratio, which is = 0.4. Figure 10 shows the NDI for all scenarios under a truckload at the speed of 2.5 m/s in a noisy environment. In Figure 10, the solid lines are the noise-free NDIs, and the dashed lines are the noisy NDIs with an RMS ratio = 0.4.

As is seen in Figure 10, the solid line and dashed line of a scenario are almost on each other. That is because the QRT is inherently a de-noising technique, and therefore, the resulting QRT-based methods are also robust to noise. Since the results in a highly noisy environment (RMS ratio = 0.4) are good, the results of acceleration responses with lower noises are not reported here.

### 5.4. Quantifying the Severity of Damage

**Figure 11:**
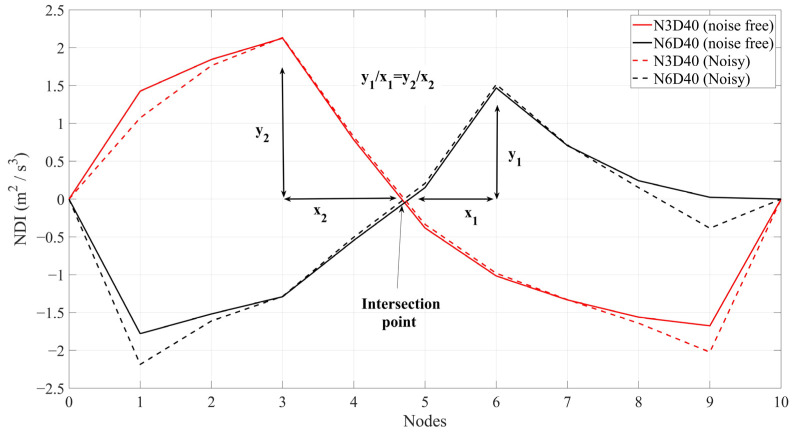
Acceleration response at Node 3 under a truckload with a speed of 2.5 m/s. Solid lines are noise-free responses, and dashed lines are noisy responses (RMS ratio = 0.4).

The approach used in [33] is employed here to quantify the severity of structural damage. It should be noted that only single-damage scenarios are discussed here in this subsection. A closer look at Figure 7 and Figure 10 shows an intersection point of the lines between Nodes 4 and 5 where all DIs have a 0 value. That means the proposed method is incapable of either locating the damage or quantifying the severity of damage in the vicinity of the middle of the bridge. Figure 11 shows the intersection point and the concept of damage quantification using DIs.

As can be seen from Figure 11, the relative slope between the place of damage and the intersection point is the same for the same single-damage scenarios. Since the high level of white noise can hardly change the curve of DIs, noise will not affect the results of damage quantification either. On the other hand, the values of DIs at the maximum of the curves are almost the same between noisy and noise-free DIs. It was mentioned that the acceleration responses were obtained at uniformly distributed nodes along the bridge. The bridge is 25 m and there are 9 nodes; hence, each node is 2.5 m from the next node. Therefore, the exact point of the intersection is 12.09 m. For simplicity in the calculation, the intersection is considered to be at a distance of 12 from the left of the bridge. Based on Figure 11, the relative slope between the maximum in DIs and the intersection point can be used for damage quantification. Table 4 compares the relative slope of the different single damage scenarios with those in the work of [33].

The red color in Table 4 represents out-of-range data. Hence, the proposed method in this paper only has one out-of-range item of data, which is in the N3D30, with a velocity of 8 m/s. As reported in Table 4, the results of the QRT-based proposed method are more accurate than the SGF-based bridge health monitoring method explained in [33]. Therefore, using the QRT-based trendline can increase the calculation’s accuracy compared with the Savitzky–Golay filter. Based on Table 3, the relative slope equal to 0.26 represents 30% of the damage, and the relative slope equal to 0.5 represents 40% of the damage in all scenarios. It should be noted that the noisy data with an RMS ratio = 0.4 were used to calculate the data in Table 4.

### 5.5. Baseline Estimation

**Figure 12:**
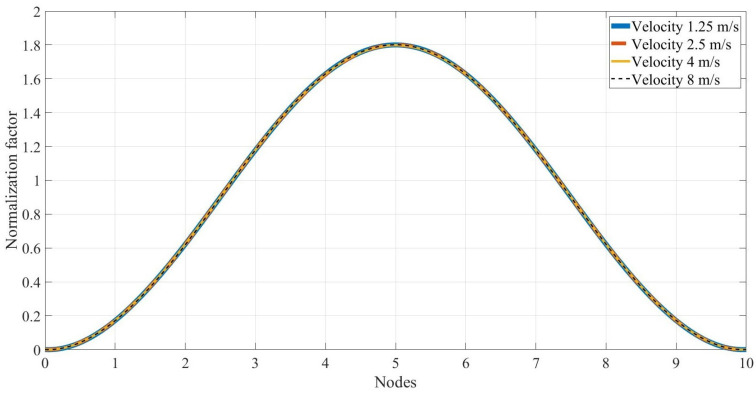
The normalization factor of all nodes along the bridge.

It was clearly shown that the normalized energy-based damage index used for this paper can accurately identify the location and severity of the structural damage. It should be noted that the normalization factor is a critical factor for the proposed method. In fact, this paper’s normalization factor (Equation (7)) plays the role of the baseline for the proposed method. The noisy data with an RMS ratio = 0.4 are used, and the normalization factors for all nodes are calculated and listed in Table 5. The normalization factors were only calculated for the undamaged scenario. Figure 12 shows Table 5 in the curve forms.

As seen in Figure 12, the normalization factor of all nodes follows a Gaussian pattern. Moreover, all the curves belonging to different velocities fall on each other. Hence, based on Figure 12, three significant advantages can be learned, namely: (1) it is acceptable to use the normalization factor of a velocity for another velocity, and that means there is no need to keep the vehicle velocity constant during the tests, (2) if an accelerometer experiences a problem or there is no accelerometer at a position, it is easy to calculate its normalization factor using the Gaussian pattern, (3) there is no need to have a uniformly distributed accelerometer for the undamaged scenario and the Gaussian pattern can be easily determined using only a few accelerometers. Therefore, since the baseline can be easily calculated using the Gaussian pattern and the proposed method can be considered a baseline-free method.

## 6. Conclusions

The main goal of this paper was to study the application of the quadratic regression technique to determine a special trendline for bridge health assessment. These special trendlines were then used to propose an output-only damage-detection method for bridge-health monitoring. It was shown that an energy-based damage index calculated from acceleration trendlines can accurately identify the location and severity of damage in the bridge. A numerical model of a 25 m simply supported bridge under a truckload was utilized to provide numerical proof for the proposed method. It was shown that, for the velocity up to 4 m/s, the structural damage location and severity can be successfully identified. The damage identification for a velocity of 8 m/s has some margin of error.

There are some excellent advantages of the proposed method that are briefly listed below:

1. The quadratic regression technique is a low-pass filter and denoising tool, and hence, its resulting damage-detection method is also robust to noise;
2. A high noise level with RMS ratio = 0.4 was used to show that the proposed method can locate and quantify the bridge structural damage;
3. Fitting a Gaussian curve on the normalization factor helps us to guess the baseline for other velocities and positions not reported in this paper;
4. The proposed method can identify the damage in a multi-damage scenario.

In the end, it should be noted that the proposed method only uses acceleration data. The proposed method does not need to monitor the dynamic modal properties or have prior knowledge about the damage location and severity. Since the proposed method has a very flexible baseline estimation, it does not need to keep the vehicle velocity the same during the tests. That means we can use a unique baseline for tests with different damage scenarios and velocities up to 4 m/s. Hence, the proposed method is categorized as an output-only, baseline, and quick bridge damage-detection method. In the future, we will try to bring this method into real practice, providing some real field data to support the proposed method in the field.

## Figures and Tables

**Table 1:** Properties of the simply supported beam.

Properties	Unit	Symbol	Value
Length	m	L	25
Mass per Unit	kg/m	μ	18,360
Stiffness	$Nm^{2}$	EI	4.865 × 10^10^

**Table 2:** Properties of the moving sprung mass.

Properties	Unit	Symbol	Value
Body mass	kg	$m_{b}$	16,500
Axle mass	kg	$m_{t}$	700
Suspension stiffness	$Nm^{-1}$	$K_{s}$	8 × 10^5^
Suspension damping	$Nm^{-1}$	$C_{s}$	2 × 10^4^
Tire stiffness	$Nm^{-1}$	$K_{t}$	3.5 × 10^6^
Velocity	m/s	V	1.25, 2.5, 4, 8

**Table 3:** Five damage scenarios considered for this study.

Scenario	1	2	3	4	5
Crack depth to the beam height ratio	30%	40%	30%	40%	40%
Location	At Node 3	At Node 6	At Node 3	At Node 6	At Nodes 3 and 6
Name	N3D30	N3D40	N6D30	N6D40	N3N6D40

Note: The scenarios are designated with N (damage location) and D (ratio).

**Table 4:** Relative slope corresponding to different velocities of single damage scenarios.

Scenario	Velocity (m/s)	Slope		Scenario	Velocity (m/s)	Slope	
		The Proposed Method	Method in [32]			The Proposed Method	Method in [32]
N3D30	1.25	0.26	0.31	N3D40	1.25	0.47	0.52
	2.5	0.26	0.34		2.5	0.47	0.47
	4	0.28	0.06		4	0.47	0.33
	8	0.20	−0.24		8	0.51	0.20
N6D30	1.25	0.26	0.32	N6D40	1.25	0.51	0.55
	2.5	0.26	0.25		2.5	0.51	0.46
	4	0.30	0.23		4	0.56	0.52
	8	0.26	0.47		8	0.50	0.81
Average		0.26	0.27	Average		0.50	0.49

**Table 5:** Normalization factor, $\gamma_{i}$, for all four velocities.

Velocity	Node 1	Node 2	Node 3	Node 4	Node 5	Node 6	Node 7	Node 8	Node 9
1.25	0.1715	0.6220	1.1780	1.6286	1.8003	1.6286	1.1781	0.6214	0.1715
2.5	0.1723	0.6218	1.1788	1.6253	1.8006	1.6286	1.1785	0.6222	0.1720
4	0.1723	0.6222	1.1788	1.6269	1.7988	1.6277	1.1785	0.6226	0.1723
8	0.1708	0.6189	1.1792	1.6292	1.8013	1.6284	1.1780	0.6219	0.1722

## Data Availability

The data presented in this study are openly available in Researchgate at [doi:10.13140/RG.2.2.19174.16962], reference number [43].
